# Determinants of Night‐Shift Scheduling Quality for Nurses in Korea: A Multilevel Approach

**DOI:** 10.1155/jonm/9937826

**Published:** 2026-04-24

**Authors:** Kyongok Park, Juyeon Oh, Jiwon An, Young Soo Chu, Hyeon Sik Chu

**Affiliations:** ^1^ Department of Nursing, Kangwon National University, 150 Namwon-Ro Heungeop-Myeon, Wonju-Si, 26403, Gangwon-Do, South Korea, kangwon.ac.kr; ^2^ College of Nursing, Dankook University, 119 Dandae-Ro Dongnam-Gu, Cheonan-Si, 31116, Chungcheongnam-Do, South Korea, dankook.ac.kr; ^3^ Department of Nursing, Far East University, 76-32 Daehak-Gil Gamgok-Myeon, Eumseong-Gun, 27601, Chungcheongbuk-Do, South Korea, fec.edu.tw; ^4^ Korea University Anam Hospital, 73 Goryeodae-ro Seongbuk-gu, Seoul, 02841, South Korea, kumc.or.kr

## Abstract

**Background:**

Night‐shift work burdens nurses and increases the likelihood of medical errors. This study aims to identify the factors that influence the quality of night‐shift scheduling.

**Methods:**

This study is framed as a secondary data analysis. Original data are drawn from nurses’ roster schedules and questionnaires completed by unit managers. The dataset had a nested structure comprising 1566 nurses within 81 wards. Multilevel analysis was conducted to examine factors influencing the quality of night‐shift scheduling at both the individual and organizational levels.

**Results:**

The majority of participants were female (95.7%, *n* = 1498). The night‐duty RN‐to‐patient ratio was 8.83 (SD = 2.34). The mean overall quality of night‐shift scheduling among participants was 2.90 (SD = 0.35). In Model 2, which included both individual‐ and organizational‐level variables, the only statistically significant individual‐level predictor of the quality of night‐shift scheduling was gender (*β* = – 0.88, *p* = 0.032). At the organizational level, the number of night‐shift‐only RNs (*β* = 0.24, *p* = 0.001), ward type (*β* = 0.12, *p* = 0.026), and the night‐duty RN‐to‐patient ratio (*β* = 0.25, *p* = 0.002) were significant.

**Conclusion:**

The quality of night‐shift scheduling was greater for female nurses, Comprehensive Nursing and Care Service (CNCS) wards supported by governmental policy, wards with an RN‐to‐patient ratio of more than 8 during night shifts, and wards with more night‐shift‐only RNs. Therefore, these results indicate that the composition and number of nursing staff in a ward contribute to improving the working conditions and environments of nurses’ shift work.

**Nursing Implications:**

The higher quality of night‐shift scheduling observed in CNCS wards and wards run on the basis of the policy of night‐shift‐only RNs suggests that governmental support positively influences individual nurses’ shift work environments. These findings support the need for policy‐level interventions to improve the quality of shift work.

## 1. Introduction

Nurses constitute the largest segment of healthcare workers and are essential contributors to the healthcare system. To maintain the 24‐h operation of healthcare institutions, nurses must engage in shift work, which is defined as “working nonstandard hours” [1]. This approach often includes early work starts, compressed workweeks with 8 or 12 h shifts, and night work. Night shifts, in particular, pose significant challenges for nurses, as they disrupt natural circadian rhythms [2]. Given the critical need for uninterrupted patient care, night shifts remain an unavoidable aspect of nursing practice despite their inherent challenges. Nursing is inherently a physically and mentally demanding profession, and shift work intensifies these demands, leading to disturbed sleep, fatigue, and increased risks of both acute and chronic health conditions [3]. Furthermore, shift work can result in emotional burdens, such as depression, anxiety, and burnout, which contribute to reduced nursing performance, including an increased likelihood of medical errors, presenteeism, and turnover, thereby jeopardizing patient safety [4].

Poorly designed work scheduling patterns exacerbate the difficulties that nurses face in adapting to shift work, significantly increasing their risks of fatigue, stress, and burnout [5]. Extensive research has documented the relationship between shift scheduling patterns and nurses’ health outcomes. Studies have shown that the number of night and evening shifts per month is positively associated with acute and chronic fatigue [6]. Frequent night shifts negatively affect one’s sleep quality, leading to fragmented nighttime sleep and insufficient daytime recovery [7]. In addition, an increased frequency of night shifts is linked to higher levels of anxiety and stress [8]. This burden is further exacerbated when the remaining periods between shifts are inadequate. For example, insufficient rest time has been shown to significantly correlate with increased fatigue [9]. A previous study reported that nurses who work rotating night shifts with quick returns experience significantly greater lethargy, exhaustion, and weariness than those who work only day shifts do [10]. Moreover, quick return schedules are strongly associated with shorter sleep duration, heightened sleepiness, and fatigue [11, 12].

It is therefore imperative to develop scheduling that enables nurses to balance their work and personal lives while ensuring that they receive adequate rest and recovery periods after shifts. This is especially important for night‐shift workers, as the day following a night shift is predominantly spent on sleep and recovery, thereby consuming more than half of the 24‐h cycle [13]. Recognizing these challenges, many countries have introduced policies, regulations, and guidelines to help optimize nurses’ work schedules [3, 14, 15].

In 2019, the Ministry of Health and Welfare of Korea launched guidelines for night shifts for hospital nurses [16, 17]. The guidelines have been evaluated positively in terms of improving the working environment during night shifts through economic incentives for hospitals [18]. These guidelines recommend limiting night shifts to no more than seven per month, restricting consecutive night shifts to a maximum of three, and ensuring a minimum rest period of 48 h following two or more consecutive night shifts. These guidelines include not only provisions on night‐shift scheduling practices but also measures for adjusting workload during night shifts, implementing special health examinations for nurses engaged in night‐shift work and prohibiting participation in education programs and organizational events following night duty. In 2022, the Ministry of Health and Welfare launched a pilot program to improve the rotating shift system for nursing, and the instructions for this program incorporated major components of the 2019 night‐shift guidelines in relation to night‐shift work [19]. The 2022 Nurse Shift System Improvement Pilot Program proposed eight evaluation criteria for shift‐work scheduling, including the three items from the 2019 Night‐Shift Work Guidelines. In this national program, the quality of shift‐work scheduling was evaluated as the percentage of the eight criteria met by each individual nurse, indicating the level of compliance with the program’s scheduling standards.

In addition, the Ministry developed the Comprehensive Nursing and Care Service (CNCS) to help overcome these challenges [20]. The CNCS is a government‐supported program designed to improve nursing staffing levels by linking hospital reimbursement to nurse‐to‐patient ratios. Wards participating in the CNCS are required to meet higher staffing standards, which may facilitate better compliance with recommended shift schedules and enhance nurses’ capacity to deliver continuous and high‐quality care. By ensuring adequate professional nursing coverage, the CNCS aims to improve the quality of patient care, increase patient satisfaction, and reduce the burden of caregiving on families.

The CNCS represents a significant step forward in improving the quality of healthcare in Korea. As services continue to evolve, they are expected to play an increasingly important role in meeting the healthcare needs of the country’s growing elderly population. Another positive evaluation is that this service presents a proper staffing ratio on the basis of the number of patients per nurse by shift [21]. While these guidelines and programs are not fully mandated by law, they are strongly supported through financial incentives and policy monitoring. The Nursing Act enacted in 2024 further emphasizes the importance of improving nurses’ working conditions, including shift work environments, although detailed regulations for night‐shift scheduling continue to evolve.

Extensive studies regarding shift work for nurses have been conducted to identify the relationship between shift work and health. Several studies have focused on shift‐work patterns or the quality of shift work. However, to our knowledge, no studies have specifically examined the factors associated with the quality of night‐shift scheduling. Therefore, the goal of the current study is to analyze the characteristics of shift nurse schedules, with a particular emphasis on night shift patterns, and to identify the factors that influence the quality of night‐shift scheduling. Specifically, a multilevel analysis is employed to examine both the individual and unit factors (organizational level) that influence night‐shift schedule quality.

## 2. Methods

### 2.1. Design and Data Collection

This study is framed as a secondary data analysis. The data used in the present study were extracted from the primary data collected for “A Study on the Improvement of Shift Work for Nurses Working in Hospitals” [22]. The primary data were collected from January 29 to March 20, 2024. The current study includes information on 1566 nurses and 81 wards reported by unit managers. This study was granted an exemption from review by the institutional review board of the researcher’s affiliated institution (IRB No. GWNUIRB‐R 2025‐5).

### 2.2. Measurements

The current study focused on roster schedules for nurses working across three shifts. Therefore, nurses who worked only one shift, two shifts for 12 h, or night shifts were excluded. Among the shift‐work roster schedules for the three months ranging from September to November 2023 available in the original data, only the shift‐work schedules for November were analyzed. Because September and October include many holidays, such as Chuseok and the National Foundation Day, which could lead to unusual work schedules, we conducted our analysis using only the November schedules. The original data were collected by unit managers. Unit managers submitted the shift‐work schedules for inclusion in the research dataset. During the process of collecting the original data, unit managers anonymized the nurses’ names, such as Nurse 1, Nurse 2, and Nurse 3, to protect the personal information of individual nurses. The shift‐work roster included information on each nurse’s age, education level, clinical experience, and job position as individual‐level variables. The number of RNs, beds, and night‐shift‐only nurses; type of ward or hospital; and the RN‐to‐patient ratio during night shifts were extracted from the original data as organizational‐level variables.

The term “night shift” refers to working from 22:00 to 06:00, with slight variations of approximately ±1 h depending on hospital policies [16]. Night‐shift‐only nurses refer to nurses who exclusively work night shifts. The wards were categorized into CNCS wards and other general wards.

The wards included in previous studies were predominantly affiliated with tertiary or general hospitals with more than 500 beds. In South Korea, a general hospital is defined as a medical institution with at least 100 beds and medical specialists available in each department. Tertiary hospitals specialize in treating critically ill patients and handling complex and highly difficult medical cases. They are designated by the Ministry of Health and Welfare and must meet specific criteria to qualify as tertiary general hospitals. As of 2024, there are approximately 45 tertiary general hospitals in Korea. Most of these hospitals are university‐affiliated hospitals [23]. For the RN‐to‐patient ratio during the night shift, the number of RNs and inpatients during the night shift was extracted from the shift schedules.

This study defines night shift quality in terms of scheduling, such as frequency and postshift rest, rather than workload. Three items used to assess the quality of night‐shift scheduling were extracted from the original data. The three items used to identify the quality of shift work were created according to the 2019 guidelines for night shifts for hospital nurses and the 2022 Pilot Project to Improve Shift Work [16, 19]. The 2022 Pilot Project sought to enhance nurses’ working conditions by introducing more predictable and efficient shift arrangements. The eight items for evaluating the quality of shift work were developed through the activities of the Pilot Project Performance Evaluation Committee, which consists of experts and representatives recommended by relevant organizations, thereby supporting the validity of the overall index [19].

The three items were as follows: Capping the number of monthly night shifts at seven, limiting the number of consecutive night shifts to a maximum of three, and mandating a minimum 48‐h rest period following two or more consecutive night shifts. In accordance with the evaluation framework of the 2022 Nurse Shift System Improvement Pilot Program, each item was coded as 1 if the guideline was met and 0 if it was not met. The quality of night‐shift scheduling was calculated as the sum of these three items, yielding a total score ranging from 0 to 3, with higher scores indicating better scheduling quality.

### 2.3. Data Analysis

The data were analyzed using IBM SPSS Statistics for Windows, Version 28.0 (IBM Corp., Armonk, NY, USA), and Stata statistical software, Version 18 (StataCorp LLC, College Station, TX, USA). The general characteristics of individuals and organizations, as well as the quality of night‐shift work patterns, were analyzed in terms of frequency, percentage, mean, and standard deviation.

To examine the factors influencing the quality of night‐shift scheduling, multilevel analysis was conducted using a random intercept model. Specifically, a two‐level linear mixed‐effects model with random intercepts at the organizational (ward) level was fitted using Stata. To ensure stable estimation in the multilevel model, wards with fewer than 10 nurses and cases with missing values on individual‐level variables were excluded. As a result, a total of 76 wards and 1349 nurses were included in the final analysis.

This method allows for the examination of the influences of both individual‐ and organizational‐level variables on an individual‐level dependent variable, with random intercepts accounting for variability at the organizational level. In the current study, the use of both individual‐level and organizational‐level variables was deemed appropriate for explaining the quality of night‐shift scheduling for nurses. Thus, a null model containing only the intercept (and no independent variables) was first constructed to determine whether the organizational‐level variance was significant, thus confirming the need for multilevel analysis.

Two models were subsequently constructed: Model 1, which included only individual‐level predictors, and Model 2, which included both individual‐ and organizational‐level predictors. Model fit was assessed via log‐likelihood, Akaike information criterion (AIC), and Bayesian information criterion (BIC) values, and fixed effects at both the individual and organizational levels were analyzed. All the statistical tests were two‐tailed and conducted at the 0.05 level of significance.

## 3. Results

### 3.1. Descriptive Statistics

Table 1 presents the individual characteristics of the nurses and the organizational‐level characteristics examined in this study. The majority of the participants were female (95.7%, *n* = 1498). With respect to clinical experience, nurses with less than 3 years of experience constituted the largest group (51.1%, *n* = 776), followed by those with 5 or more years (27.0%, *n* = 411) and those with 3–5 years (21.3%, *n* = 333). In terms of educational background, most of the participants held an associate’s or bachelor’s degree in nursing (94.3%, *n* = 1441). Moreover, the majority of the participants worked as staff nurses (88.5%, *n* = 1368).

**TABLE 1:** General characteristics of individual and organizational factors (individual *N* = 1,566, organization *N* = 81).

Level	Variables	Categories	*n* (%) or *M* ± SD
Individual	Gender	Male Female	68 (4.3) 1498 (95.7)
	Clinical experience^†^	< 3 years 3–5 years > 5 years	776 (51.1) 333 (21.3) 411 (27.0)
	Education level^†^	ADN/BSN Postgraduate or higher	1441 (94.3) 87 (5.7)
	Job position	Charge nurse Staff nurse	180 (11.5) 1368 (88.5)
	Quality of night‐shift scheduling	1 2 3	2.90 ± 0.35 25 (1.6) 114 (7.3) 1427 (91.1)

Organizational	Number of RNs	< 20 20–40 > 40	29.67 ± 10.55 11 (14.7) 51 (68.0) 13 (17.3)
	Number of beds	< 35 35–45 > 45	45.00 ± 9.70 8 (10.7) 35 (47.6) 32 (42.7)
	Number of night‐shift‐only nurses	None 1‐2 ≥ 3	25 (33.3) 23 (30.7) 27 (36.0)
	Type of ward	General ward CNCS ward	49 (65.3) 26 (34.7)
	Night‐duty RN‐to‐patient ratio^†^	≤ 8.0 > 8.0	8.83 ± 2.34 24 (34.0) 46 (65.7)
	Type of hospital	Tertiary hospital General hospital	58 (77.3) 17 (22.7)

Abbreviations: ADN, associate degree in nursing; BSN, Bachelor of Science in Nursing; CNCS, Comprehensive Nursing and Care Service; quality of night‐shift scheduling = sum of three binary items (range 0–3); RN, registered nurse.

^†^Missing cases were excluded.

The mean number of RNs per ward was 29.67 (SD = 10.55), with wards comprising between 20 and 40 RNs being the most common (68%, *n* = 51). The mean number of beds per ward was 45 (SD = 9.70), and wards with 35–45 beds were predominant (47.6%, *n* = 35). With respect to the number of night‐shift‐only nurses, wards with three or more RNs, who were night‐shift‐only nurses, were the most common (36%, *n* = 27), followed by wards with no RNs on fixed night duty (33.3%, *n* = 25) and those with 1–2 RNs on fixed night duty (30.7%, *n* = 23). Among these units, general wards accounted for 65.3% (*n* = 49), whereas CNCS wards represented 34.7% (*n* = 26). The night‐duty RN‐to‐patient ratio was 8.83 (SD = 2.34); wards with a ratio exceeding 8 represented 65.7% of the total (*n* = 46), whereas those with a ratio of 8 or less accounted for 34.0% of the total (*n* = 24). The number of tertiary hospitals was 58 (77.3). The majority of participants worked at tertiary hospitals.

### 3.2. The Quality of Participants’ Night‐Shift Work Patterns

**FIGURE 1:**
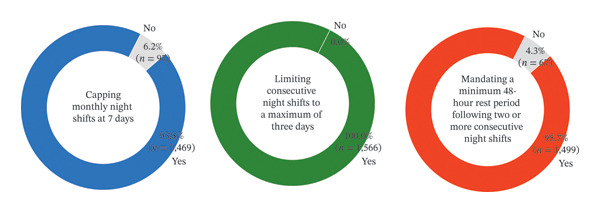
Item‐level descriptive statistics of the quality of night‐shift scheduling criteria (individual level, *N* = 1566). The values represent the proportion of individual nurses meeting each scheduling criterion.

The results regarding the quality of the participants’ night‐shift work are presented in Figure 1. The overall quality of night‐shift scheduling among the participants had a mean score of 2.90 out of 3.00 (SD = 0.35). Among the items examined within this measure, 93.8% (*n* = 1469) of the participants agreed with “capping the number of monthly night shifts at 7 days,” whereas 100% agreed with “limiting the number of consecutive night shifts to a maximum of three.” Moreover, 95.7% (*n* = 1499) agreed with “mandating a minimum 48‐h rest period following two or more consecutive night shifts.”

### 3.3. Factors Influencing the Quality of Participants’ Night‐Shift Scheduling

The results of the multilevel analysis of the quality of nurses’ night‐shift scheduling are presented in Table 2. In this study, the null model, the individual model, and the individual‐organizational model were sequentially tested. The individual‐level variables consisted of gender, clinical experience, educational level, and job position, whereas the organizational‐level variables consisted of the number of night‐shift‐only nurse RNs, the type of ward, and the night‐duty RN‐to‐patient ratio.

**TABLE 2:** Multilevel regression analysis of factors influencing the quality of night‐shift scheduling (Level‐1, *n* = 1349; Level‐2, *n* = 67).

Parameter		Variables		Model 0		Model 1		Model 2	
				Null model		Individual model		Individual‐organizational model	
				*B*	*p*	*B*	*p*	*B*	*p*
Fixed effect	Level‐1	Intercept		2.88	< 0.001	2.880	< 0.001	2.53	< 0.001
		Gender (ref: female)	Male			−0.06	0.119	−0.88	0.032
		Clinical experience (ref: < 3 years)	3–5 years > 5 years			−0.18 0.00^†^	0.385 0.985	−0.15 0.01	0.494 0.677
		Education level (ref: ADN/BSN)	Postgraduate or higher			0.04	0.301	0.03	0.458
		Job position (ref: staff nurse)	Charge nurse			0.02	0.583	0.01	0.801
	Level‐2	Number of night‐shift‐only nurses (ref: none)	1‐2 ≥ 3					0.14 0.24	0.055 0.001
		Type of ward (ref: general ward)	Comprehensive Nursing and Care Service ward					0.12	0.026
		Night‐duty RN‐to‐patient ratio (ref: < 8 patients)	≥ 8 patients					0.25	0.002

Random effect		Level‐1, *δ* ^2^ Level‐2, *μ*0(*τ*) *χ* ^2^ (*p*) ICC (%)		598.18 (< 0.001) 41.44		0.06 558.17 (< 0.001) 40.88		0.05 0.08 421.58 (< 0.001) 35.26	

Model fit Log‐likelihood AIC BIC				−299.05 604.10 620.17		−321.48 658.96 701.37		−315.43 654.86 717.34	

Abbreviations: ADN, associate degree in nursing; AIC, Akaike information criterion; BIC, Bayesian information criterion; BSN, Bachelor of Science in Nursing; ICC, intraclass correlation coefficient; ref, reference.

^†^0.0003889 (results rounded up to the fourth digit after the decimal point).

In the null model (Model 0), which contained no explanatory variables, the between‐organization variance was estimated to determine whether there were organizational differences in the quality of night‐shift scheduling. An examination of the random effects in this basic model revealed that the Level‐2 (organizational‐level) variance in the quality of night‐shift scheduling was statistically significant (*χ* ^2^ = 598.18, *p* < 0.001), indicating that meaningful variance exists at the organizational level. According to the intraclass coefficient (ICC), 41.44% of the total variance was attributable to differences among organizations.

Model 1 presents the results of analyzing the fixed effects of individual‐level variables on the quality of night‐shift scheduling. None of the individual‐level variables had a statistically significant effect on the quality of night‐shift scheduling.

In Model 2, which included both individual‐ and organizational‐level variables, the only statistically significant individual‐level predictor of the quality of night‐shift scheduling was gender (*β* = −0.88, *p* = 0.032). At the organizational level, the number of night‐duty‐only RNs (*β* = 0.24, *p* = 0.001), ward type (*β* = 0.12, *p* = 0.026), and the night‐duty RN‐to‐patient ratio (*β* = 0.25, *p* = 0.002) were significant. After both individual‐ and organizational‐level variables were included in Model 2, the ICC was 35.26%, indicating that the unexplained variance at the organizational level decreased compared to the ICC of 40.88% found for Model 1.

## 4. Discussion

### 4.1. The Quality of Night‐Shift Scheduling

The current study was conducted with the aim of identifying the quality of night‐shift scheduling and what factors influence that quality. The findings revealed that the quality of night‐shift scheduling for nurses working three consecutive night shifts ranged from 1 to 3 and was 2.9. This score is considered high because the items reflect basic recommendations for night‐shift scheduling.

According to a previous study that analyzed data from the Korean Working Conditions Survey (KWCS), Korean nurses worked night shifts for an average of 7.14 days per month in 2014, 6.48 days per month in 2017, and 6.33 days per month in 2020 [24]. Another study reported an average number of 5.73 days per month that nurses work night shifts [18]. Cho and her colleagues suggested that the percentage of consecutive night shifts that exceed 3 days is 99.5% in Korea. Therefore, most nurses in Korea probably worked night shifts 5–7 days per month, usually in blocks of no more than two or three consecutive nights.

The compliance rate for the item “mandating a minimum 48‐h rest period following two or more consecutive night shifts” was 95.7% in the present study. However, one previous study reported a 68%–78.1% compliance rate [18]. Another study that explored compliance with items for night shifts, such as “having at least two days off after working a night shift,” reported relatively low compliance levels among 17 recommendations regarding work schedules for shift work [25]. There are compliance gaps between the findings of previous studies and those of the present study. Most of the participants in the present study were drawn from superior general hospitals, such as tertiary hospitals. There is a large gap in working conditions between superior general hospitals, such as tertiary hospitals and general hospitals [26]. Therefore, further studies are needed to confirm compliance with the item “mandating a minimum 48‐h rest period following two or more consecutive night shifts.” The indicators used to operationalize night‐shift scheduling quality primarily reflect minimum statutory requirements within the Korean healthcare system [16, 19] and thus capture institutional compliance rather than the full scope of scheduling quality. This may have resulted in restricted variability across hospitals, limiting the explanatory power of multilevel factors. Given that scheduling quality is a multidimensional construct, future research should incorporate additional dimensions such as skill mix and staffing adequacy.

### 4.2. Gender

Gender was found to influence the quality of night‐shift scheduling in the present study. The quality of night‐shift scheduling was lower for male nurses. When the Night Work Women Convention was held in 1948, the purpose of developing a recommendation for night‐shift work was to prohibit night work for women and young people. However, the international recommendation for night‐shift work changed in the early 1990s because the scope of the recommendation, such as protecting both genders equally, was extended [27]. The labor environment has changed, and working at night is no longer performed only by workers of a particular gender or age.

A previous study reported that workers who engage in night shifts are younger and more likely to be male than female [26, 28]. A previous study reported that 68% of male nurses work on fixed night shifts and 74% of male nurses work on rotating shifts, including night shifts, whereas 32% of female nurses work on fixed night shifts and 26% of female nurses work night shifts [29].

In terms of the demographic characteristics of nurses in Korea, the majority of nurses in Korea are young women, even though the number of male nurses has recently increased [28]. Male nurses are more likely to work at night because some regulations, such as those prohibiting night work during pregnancy and just after giving birth, still exist to protect women during maternity. The findings of our present study might reflect this phenomenon. Nevertheless, male participants accounted for only 4.3% of the sample, which is lower than the national proportion of male nurses in South Korea (6.3%) [30]. The interpretation of gender‐related differences should be conducted with caution. This imbalance limits the generalizability of the findings and highlights the need for further studies with more balanced samples.

### 4.3. Types of Wards

The type of ward was found to influence the quality of night‐shift scheduling in the present study. The findings of the current study revealed that the quality of night‐shift scheduling in a CNCS ward is greater than that in a general ward. In terms of the Korean health system, hospitals that implement the CNCS are reimbursed higher nursing fees, as they maintain a higher level of nursing staff [18]. A previous study reported that the CNCS promotes a nurse staffing policy that combines financial incentives for better staffing and strict restrictions on reimbursement for poor staffing. Therefore, the CNCS successfully encourages hospitals to employ larger nursing workforces [18].

The number of nurses per ward markedly differed between CNCS wards and general wards. A previous study reported that the number of RNs in a CNCS ward is approximately twice as high as that in a general ward. The mean RN‐to‐patient ratio is 5.4 in a CNCS ward, whereas it is 8.1–8.4 in a general ward [22]. Another previous study reported that the bed‐to‐RN ratio in hospitals participating in the CNCS was much higher than that in hospitals not participating in the CNCS. The financial incentive system for reimbursement and the strict regulation of staffing ratios successfully increase the overall nurse staffing levels for hospitals participating in the CNCS. Another study reported that the total effect of employment induction on the CNCS was 3.1–6.1 times greater than that found in similar industries [31]. Although the abovementioned studies did not identify a relationship between the number of nurses and the quality of night‐shift scheduling, a ward with more RNs, such as a CNCS ward, might much more easily assign RNs to night shifts in compliance with the recommendation and might more easily assign leave to nurses as well.

A previous study in Korea addressed the relationships among the number of nurses on each shift, the number of nurses on leave, and the total number of nurses in a ward [13]. The quality of shift scheduling, including that for night shifts, requires decent work, decent leave, and a decent staffing ratio. The most important component among the three abovementioned factors is the staffing ratio. Korea does not have strict regulations concerning the nurse‐to‐patient ratio. One previous study aimed to estimate the nurse‐to‐patient ratio [32]. The median nurse‐to‐patient ratio ranged from 8.8 to 10.1 in tertiary hospitals and 9.4 to 11.0 in general hospitals [32]. These nurse‐to‐patient ratios imply very high‐intensity labor among nurses in tertiary hospitals and general hospitals in Korea. Therefore, increasing the number of hospital nurses through expanded recruitment may help improve the quality of night‐shift scheduling and reduce labor intensity.

### 4.4. The Number of Patients Per Nurse by Night Shift

The RN‐to‐patient ratio during the night shift was found to influence the quality of night‐shift scheduling. The findings of the current study revealed that having more than eight patients per nurse during night shifts increases the quality of night‐shift scheduling compared to having eight or fewer patients per nurse during night shifts. This study defines night shift quality in terms of scheduling, such as frequency and postshift rest, rather than workload. At the ward level, a smaller number of night shift staff members caring for more than eight patients may result in fewer night shifts and extended rest periods (e.g., more than 48 h) after night duty. A previous study suggested that the amount of night‐shift work per person might be reduced by a shift system that includes more daily work, with the aim of reducing health and safety risks [32]. The results of this study indicate that having fewer nurses working at night increases the quality of night‐shift scheduling. These findings suggest that if fewer nurses are assigned to work at night, then more nurses are able to take a day off or perform other shifts, such as day or evening shifts.

The ideal staffing level of the night shift is debatable. Nurses reported that staffing levels are often inadequate at night or that their workload is too high, leading them to report that the quality of care they deliver is negatively affected​ [33, 34]. A previous study reported that nurses working at night or on weekends should complete more tasks as a result of inadequate staffing levels during night shifts or weekend shifts [35]. Importantly, although higher patient‐to‐nurse ratios may appear to improve scheduling quality, they also entail significant trade‐offs. A high ratio is well known to increase workload, which in turn contributes to nurse fatigue, burnout, and medical errors, as well as poor patient outcomes [36, 37]. Therefore, both adequate staffing and scheduling quality must be considered in tandem. In our study, the ≥ 8 RN‐to‐patient ratio was identified as a key finding and should be interpreted as an indicator of scheduling quality rather than a direct measure of staffing adequacy. Ensuring sufficient total nurse staffing at the unit level is essential for maintaining safe patient‐to‐nurse ratios while also allowing for well‐designed and sustainable night‐shift schedules. Further research is needed to determine the optimal number of nurses required for night shifts, as well as the appropriate overall staffing levels per unit, to achieve both safe care delivery and effective scheduling.

### 4.5. Night‐Shift‐Only Nurses

This study revealed that the number of night‐shift‐only nurses influences the quality of night‐shift scheduling.

According to the 2019 guidelines for night shifts for hospital nurses, night‐shift workers are those nurses who work only 14 or 15 night shifts per month. If there are night‐shift‐only nurses in wards, then it is much easier to schedule shifts. Three‐shift nurses might have a lower likelihood of working night shifts primarily because night‐shift‐only nurses work at night. Therefore, having more night‐shift‐only nurses might influence the high quality of night‐shift work for the other three types of shift‐work nurses.

There are a few issues related to night‐shift‐only nurses. Working permanent night shifts is associated with health problems and social isolation [33, 38, 39]. Powell conducted qualitative research on 14 nurses working night shifts in regional Australian hospitals. Compared to day staff, night‐shift nurses reported not only strong collegial relationships but also feelings of isolation and lack of recognition. They also faced limited professional development opportunities. The participants consistently highlighted pervasive fatigue, sleep disruption, and social isolation, which had lasting effects on their personal well‐being and ability to engage fully in social and family life. While some valued the flexibility that night work provided, most described long‐term challenges to both health and career development [34]. A previous study examined the relationship between night work and sickness absence among hospital nurses using three years of electronic rostering data from 32 wards in an English hospital [38]. Analysis revealed that when the proportion of night shifts exceeded 75%, the risk of sickness absence increased significantly. These findings suggest that nurses working permanent night shifts may be more vulnerable to long‐term health problems, including chronic fatigue, musculoskeletal disorders, and mental health issues.

However, in terms of comparisons between night‐shift‐only nurses and rotating shift nurses, previous studies have reported that nurses prefer working permanent night shifts over rotating shifts [33, 35, 40]. While working night shifts is exhausting, it also gives nurses a sense of fulfillment [41], and the wards are less stressed at night, with reduced noise levels, fewer nursing task interruptions, and increased focused‐thinking time [35]. Nurses are able to care for their families during the day if they work night shifts [33]. Therefore, a proper number of night‐shift‐only nurses should be assigned to work at night in accordance with the 2019 night‐shift work guidelines and be required to undergo an occupational medical examination at least once a year [42]. According to guidelines for night shifts in Korea, night‐shift‐only work is restricted to a maximum of 3 months, although it may be extended with the employee’s consent and through a labor‐management agreement. Compliance with these regulations is essential, and even when nurses voluntarily continue night‐shift‐only work for longer periods, sufficient training opportunities, such as online education, should be provided. Furthermore, given the substantial proportion of night‐shift‐only nurses, hospitals should allocate dedicated training hours that reflect their working schedules [43]. If the abovementioned conditions, such as adherence to individual preferences and not being implemented for too long a period, are met, then night‐shift‐only nurses will continue to contribute to improving the quality of night‐shift scheduling.

## 5. Limitations

Several limitations of this study should be acknowledged. First, most hospitals included in the analysis were tertiary hospitals or large‐scale general hospitals. Compared to smaller general hospitals, these institutions tend to demonstrate higher compliance with scheduling guidelines because of greater staffing capacity and resource availability [25]. Therefore, the findings may not be generalizable to nurses working in small‐scale hospitals.

Second, in cases where ward‐level data on the number of patients per shift were unavailable, the daily patient count was used as a proxy. This assumption may have limited the precision of the estimates related to nurse‐to‐patient ratios during night shifts. In addition, owing to the nature of secondary data analysis, important individual‐level variables such as age, marital status, number of children, and sleep−wake patterns could not be included. These factors are known to influence shift work tolerance and scheduling preferences [42, 44], and future studies should incorporate such variables to provide a more comprehensive understanding of the determinants of night‐shift scheduling quality.

Third, although the overall shift‐work quality index has been validated in prior research, the three items used to assess night‐shift scheduling quality were adopted on the basis of a policy‐driven measurement framework rather than being independently validated as a standalone subscale in the present study, which represents a methodological limitation.

Fourth, the dependent variable representing night‐shift scheduling quality was derived from three binary items and ranged from 0 to 3. Although this summated score was treated as an approximately continuous outcome in the multilevel analysis, its restricted range and discrete nature may have limited the precision of the parameter estimates and reduced the statistical power, particularly for detecting organizational‐level effects. Nevertheless, the large sample size and the inclusion of 81 wards likely mitigated some of these limitations by providing a stable estimation of average effects. Relatedly, the limited improvement in information criteria such as AIC and BIC across models may be attributable to the restricted variability of the outcome, and this should be accounted for when interpreting the magnitude of fixed effects.

Moreover, one of the three components, limiting consecutive night shifts to a maximum of three, showed no variability, as all the wards met this criterion, rendering this item noninformative from a statistical perspective. While diagnostic checks did not indicate serious violations of model assumptions, the results should be interpreted as reflecting overall trends rather than precise interval‐level differences.

Finally, the gender imbalance in the sample, with a low proportion of male participants, which was also lower than the national distribution of nurses in South Korea, limits the robustness of gender‐related comparisons, and findings related to gender differences should be interpreted with caution.

## 6. Conclusion

The current study presents an investigation of the quality of night‐shift scheduling for nurses working across three shifts. The present study also aimed to identify the factors that influence the quality of night‐shift scheduling. The quality of night‐shift scheduling was lower for male nurses, those working in general wards, and those with an RN‐to‐patient ratio less than 8 during night shifts, but higher in wards with more night‐shift‐only RNs. A well‐designed shift schedule has been reported to address the health problems associated with shift work and to maintain compatibility between workers’ professional and private lives [13, 45]. CNCS and the policy of night‐shift‐only RNs are representative nursing policies in Korea. These policies appear to have positively influenced the quality of night‐shift scheduling for Korean nurses. Therefore, governmental interests and support are needed to improve the working conditions and environments of nurses’ shift work, including from the Nursing Act, which was newly enacted in 2024. While most studies on nurses’ shift work have explored the relationships between individual‐level variables and shift work, this study incorporates not only individual‐level factors but also organizational‐level variables, such as ward policies and staffing ratios, to examine how organizational factors influence individual night‐shift schedules.

## Funding

This study was funded by the Korean Hospital Nurses Association in 2023.

## Disclosure

The funding organizations played no role in the study design, data collection and analysis, decision to publish, or preparation of the manuscript.

## Ethics Statement

This secondary data analysis study, which used deidentified data with no personal identifiers, was granted an exemption from ethical review by the Institutional Review Board of Gangneung‐Wonju National University (IRB No. GWNUIRB‐R2025‐5).

## Conflicts of Interest

The authors declare no conflicts of interest.

## Data Availability

This study used secondary data from a research project funded by the Korean Hospital Nurses Association. Owing to data ownership and confidentiality agreements, the dataset is not publicly available.
